# Supplementary figures and images for: Beliefs about harms of cigarette smoking among Norwegian adults born from 1899 to 1969. Do variations across education, smoking status and sex mirror the decline in smoking?
Source: PLoS One. 2022 Aug 3;17(8):e0271647. doi: 10.1371/journal.pone.0271647 (PMC9348701; doi:10.1371/journal.pone.0271647)

Marginal change in the probability of belonging to each group

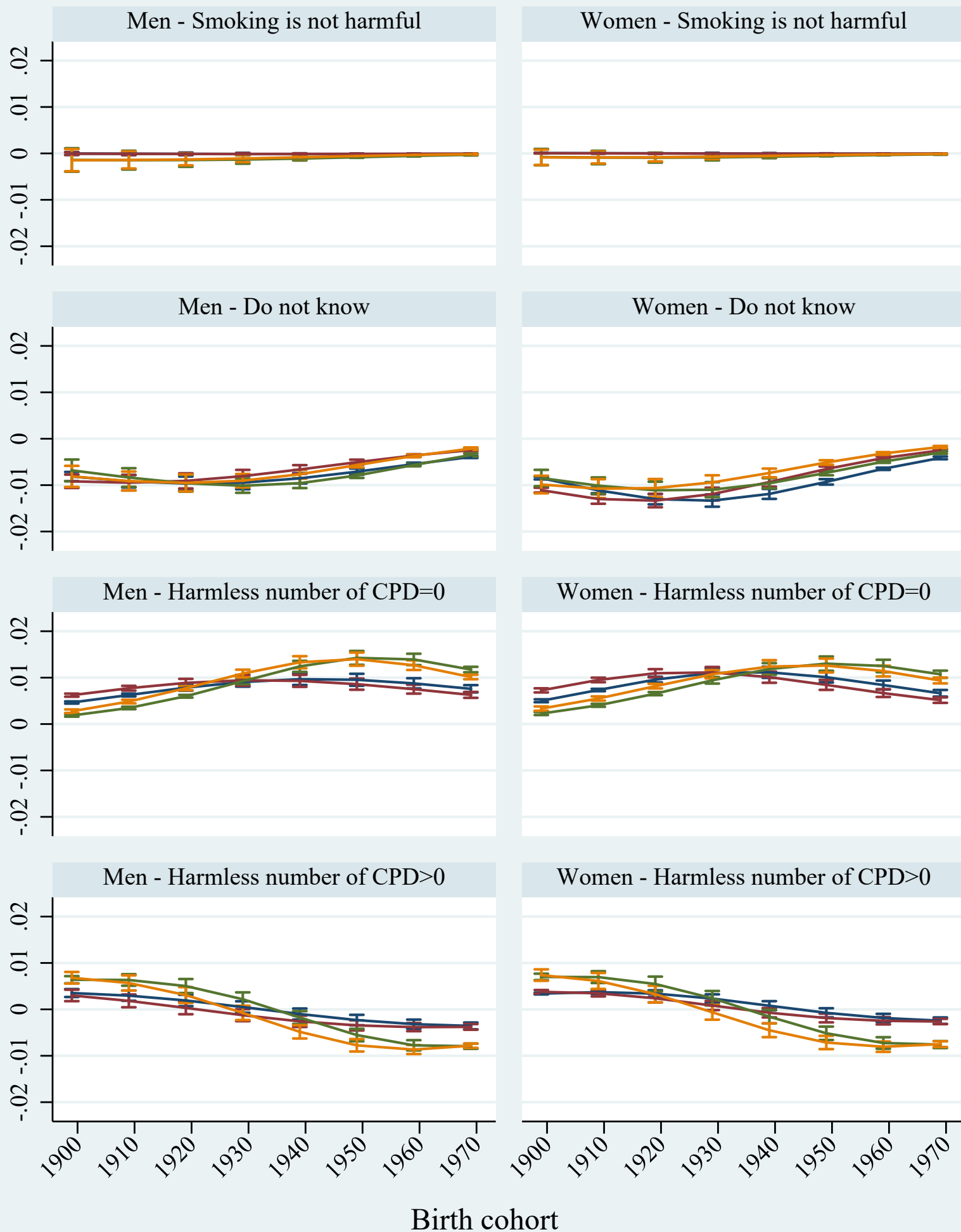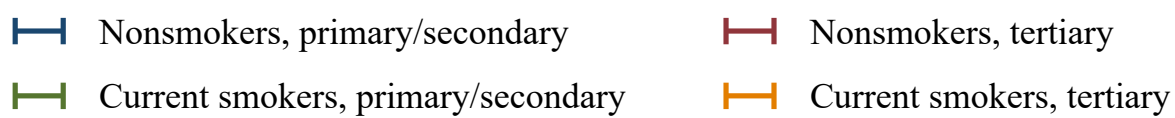

Supplement: S1 Fig — (PDF) [file pone.0271647.s001.pdf]

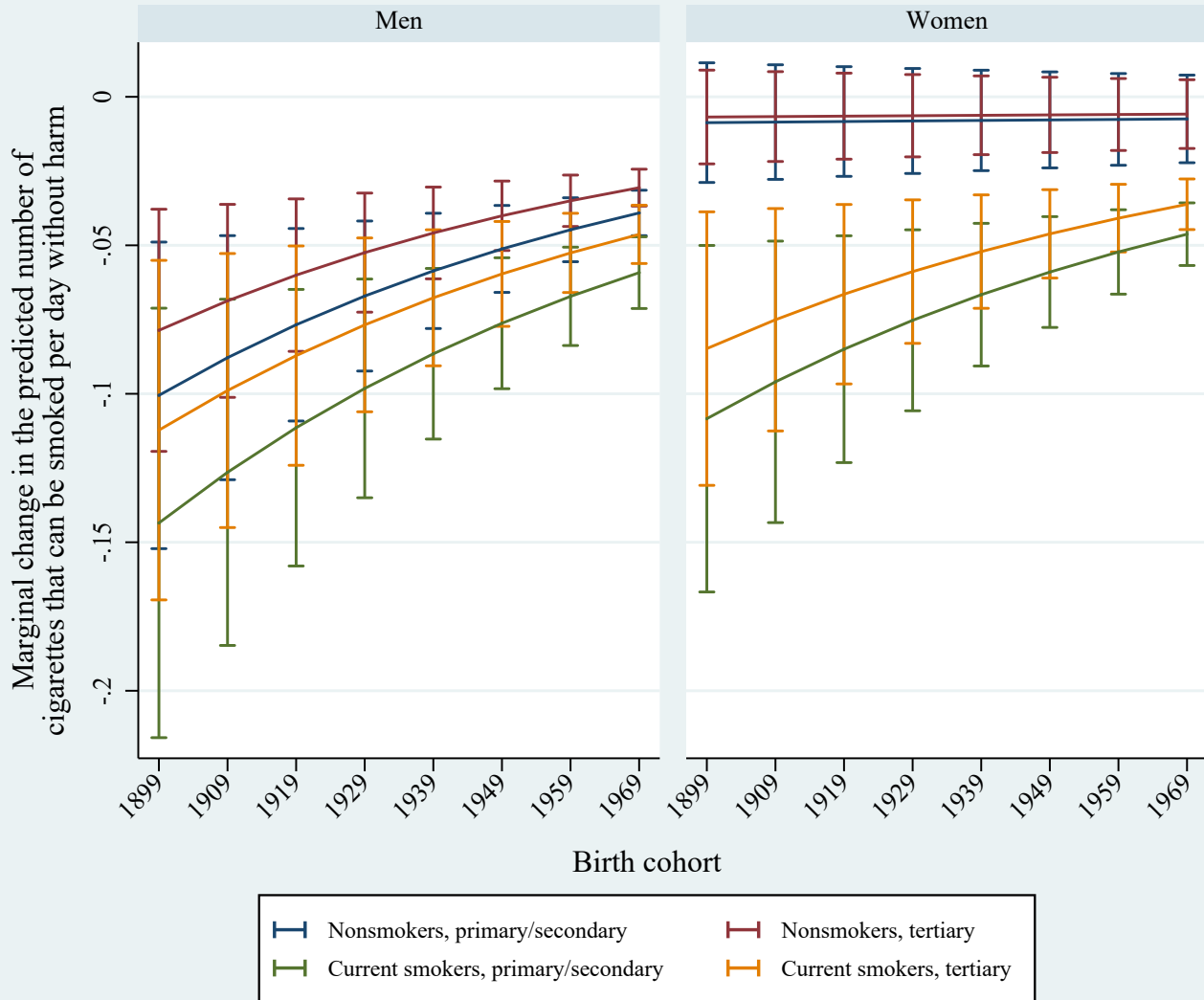

Supplement: S2 Fig — (PDF) [file pone.0271647.s002.pdf]

## Continuous age and cohort

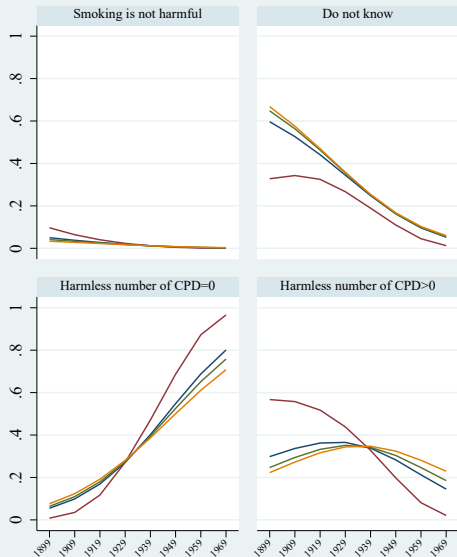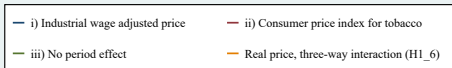

## Categorical age and cohort

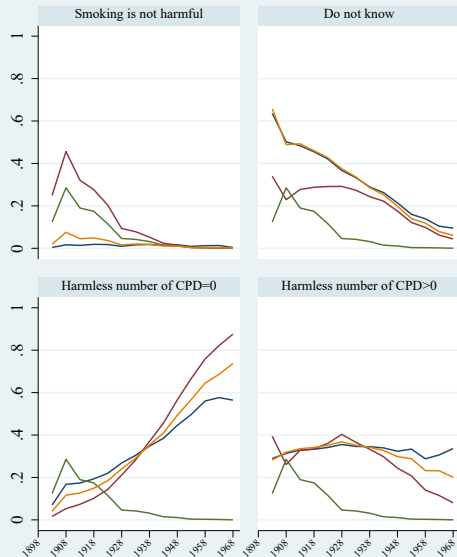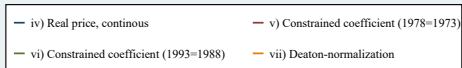

Supplement: S3 Fig — (PDF) [file pone.0271647.s003.pdf]
