# Supplementary material for: Beliefs about harms of cigarette smoking among Norwegian adults born from 1899 to 1969. Do variations across education, smoking status and sex mirror the decline in smoking?
Source: PLoS One. 2022 Aug 3;17(8):e0271647. doi: 10.1371/journal.pone.0271647 (PMC9348701; doi:10.1371/journal.pone.0271647)
Supplement: S1 Table — (PDF) [file pone.0271647.s004.pdf]

S1 Table. Regression coefficients (b), standard errors (se) and p-values (p) for models included in the first hurdle (H1)

**Outcome 1: Do not think smoking is harmful (Reference: Believe that the harmless number of CPD=0)**	Model H1_1			Model H1_2			Model H1_3			Model H1_4			Model H1_5			Model H1_6			Model H1_7		
	b	se	p	b	se	p	b	se	p	b	se	p	b	se	p	b	se	p	b	se	p
Cohort	-0.07	0.01	0.00	-0.07	0.01	0.00	-0.07	0.01	0.00	-0.04	0.01	0.00	-0.07	0.01	0.00	-0.05	0.01	0.00	-0.04	0.01	0.01
Age	-0.02	0.01	0.08	-0.02	0.01	0.09	-0.02	0.01	0.08	-0.02	0.01	0.08	-0.02	0.01	0.09	-0.02	0.01	0.08	-0.02	0.01	0.08
Period (real price)	0.06	0.02	0.01	0.06	0.02	0.01	0.06	0.02	0.01	0.06	0.02	0.01	0.06	0.02	0.01	0.06	0.02	0.01	0.06	0.02	0.01
Women*	-0.58	0.10	0.00	-17.48	13.18	0.19	-0.58	0.10	0.00	-0.54	0.10	0.00	-13.76	13.93	0.32	-17.26	25.81	0.50	-0.91	28.93	0.98
Tertiary** (reference=Primary/secondary)	-0.62	0.18	0.00	-0.62	0.18	0.00	38.33	22.68	0.09	-0.67	0.18	0.00	56.03	31.23	0.07	-0.67	0.18	0.00	70.28	57.29	0.22
Current smoker***	1.93	0.12	0.00	1.92	0.12	0.00	1.93	0.12	0.00	93.38	15.00	0.00	1.92	0.12	0.00	90.18	20.10	0.00	94.96	21.28	0.00
Eastern Norway****	0.00	0.14	0.97	-0.01	0.14	0.95	0.00	0.14	0.99	0.01	0.14	0.96	-0.01	0.14	0.96	0.01	0.14	0.97	0.01	0.14	0.96
Southern/Western Norway and Trøndelag****	-0.10	0.14	0.45	-0.11	0.14	0.43	-0.10	0.14	0.46	-0.08	0.14	0.57	-0.11	0.14	0.44	-0.08	0.14	0.56	-0.08	0.14	0.57
Northern Norway****	0.15	0.17	0.39	0.15	0.17	0.39	0.15	0.17	0.38	0.17	0.17	0.33	0.14	0.17	0.40	0.17	0.17	0.33	0.17	0.17	0.33
Women X Cohort				0.01	0.01	0.20							0.01	0.01	0.35	0.01	0.01	0.52	0.00	0.02	0.99
Tertiary X Cohort							-0.02	0.01	0.09				-0.03	0.02	0.07				-0.04	0.03	0.22
Current smoker X Cohort										-0.05	0.01	0.00				-0.05	0.01	0.00	-0.05	0.01	0.00
Tertiary X Women													-26.10	46.21	0.57				-38.04	74.58	0.61
Tertiary X Women X Cohort													0.01	0.02	0.56				0.02	0.04	0.60
Current smoker X Women																7.23	30.43	0.81	-9.15	33.42	0.78
Current smoker X Women X Cohort																0.00	0.02	0.81	0.01	0.02	0.78
Tertiary X Current smoker																			-14.25	69.04	0.84
Tertiary X Current smoker X Cohort																			0.01	0.04	0.84
Tertiary X Current smoker X Women																			30.04	98.98	0.76
Tertiary X Current smoker X Women X Cohort																			-0.02	0.05	0.76
Constant	130.64	23.03	0.00	137.24	23.48	0.00	127.02	23.10	0.00	73.95	25.51	0.00	132.47	23.56	0.00	81.20	28.24	0.00	72.10	28.94	0.01

**Outcome 2: Do not know if smoking is harmful (Reference: Believe that that the harmless number of CPD=0)**	Model H1_1			Model H1_2			Model H1_3			Model H1_4			Model H1_5			Model H1_6			Model H1_7		
	b	se	p	b	se	p	b	se	p	b	se	p	b	se	p	b	se	p	b	se	p
Cohort	-0.07	0.00	0.00	-0.07	0.00	0.00	-0.07	0.00	0.00	-0.06	0.00	0.00	-0.07	0.00	0.00	-0.06	0.00	0.00	-0.06	0.00	0.00
Age	-0.04	0.00	0.00	-0.04	0.00	0.00	-0.04	0.00	0.00	-0.04	0.00	0.00	-0.04	0.00	0.00	-0.04	0.00	0.00	-0.04	0.00	0.00
Period (real price)	0.04	0.01	0.00	0.04	0.01	0.00	0.04	0.01	0.00	0.04	0.01	0.00	0.04	0.01	0.00	0.03	0.01	0.00	0.03	0.01	0.00
Women*	-0.03	0.03	0.27	7.51	3.82	0.05	-0.03	0.03	0.28	-0.01	0.03	0.76	3.72	4.11	0.37	17.64	4.88	0.00	12.20	5.38	0.02
Tertiary** (reference=Primary/secondary)	-0.62	0.05	0.00	-0.62	0.05	0.00	3.55	6.23	0.57	-0.65	0.05	0.00	-6.16	8.35	0.46	-0.64	0.05	0.00	-8.43	10.38	0.42
Current smoker***	0.22	0.03	0.00	0.23	0.03	0.00	0.22	0.03	0.00	43.79	4.10	0.00	0.23	0.03	0.00	54.56	5.79	0.00	51.80	6.28	0.00
Eastern Norway****	0.10	0.04	0.03	0.10	0.04	0.03	0.10	0.04	0.03	0.10	0.04	0.02	0.10	0.04	0.02	0.10	0.04	0.02	0.10	0.04	0.02
Southern/Western Norway and Trøndelag****	0.14	0.04	0.00	0.15	0.04	0.00	0.14	0.04	0.00	0.16	0.04	0.00	0.15	0.04	0.00	0.15	0.04	0.00	0.15	0.04	0.00
Northern Norway****	0.21	0.06	0.00	0.21	0.06	0.00	0.21	0.06	0.00	0.22	0.06	0.00	0.21	0.06	0.00	0.21	0.06	0.00	0.22	0.06	0.00
Women X Cohort				0.00	0.00	0.05							0.00	0.00	0.36	-0.01	0.00	0.00	-0.01	0.00	0.03
Tertiary X Cohort							0.00	0.00	0.50				0.00	0.00	0.50				0.00	0.01	0.44
Current smoker X Cohort										-0.02	0.00	0.00				-0.03	0.00	0.00	-0.03	0.00	0.00
Tertiary X Women													21.13	12.66	0.10				23.18	15.13	0.13
Tertiary X Women X Cohort													-0.01	0.01	0.09				-0.01	0.01	0.12
Current smoker X Women																-19.03	8.28	0.02	-16.38	8.87	0.07
Current smoker X Women X Cohort																0.01	0.00	0.02	0.01	0.01	0.07
Tertiary X Current smoker																			16.36	17.87	0.36
Tertiary X Current smoker X Cohort																			-0.01	0.01	0.35
Tertiary X Current smoker X Women																			-3.18	28.65	0.91
Tertiary X Current smoker X Women X Cohort																			0.00	0.02	0.90
Constant	136.21	6.60	0.00	132.63	6.89	0.00	135.55	6.65	0.00	120.04	6.76	0.00	133.66	6.99	0.00	109.20	7.32	0.00	111.37	7.55	0.00

**Outcome 4:<br>Believe that safe number of CPD>0<br>(Reference: Outcome 3: Believe that the harmless number of CPD=0)**	Model H1_1			Model H1_2			Model H1_3			Model H1_4			Model H1_5			Model H1_6			Model H1_7		
	b	se	p	b	se	p	b	se	p	b	se	p	b	se	p	b	se	p	b	se	p
Cohort	-0.04	0.00	0.00	-0.04	0.00	0.00	-0.03	0.00	0.00	-0.03	0.00	0.00	-0.04	0.00	0.00	-0.03	0.00	0.00	-0.03	0.00	0.00
Age	-0.02	0.00	0.00	-0.02	0.00	0.00	-0.02	0.00	0.00	-0.02	0.00	0.00	-0.02	0.00	0.00	-0.02	0.00	0.00	-0.02	0.00	0.00
Period (real price)	0.05	0.01	0.00	0.05	0.01	0.00	0.05	0.01	0.00	0.05	0.01	0.00	0.05	0.01	0.00	0.05	0.01	0.00	0.05	0.01	0.00
Women*	-0.22	0.03	0.00	-16.32	3.60	0.00	-0.22	0.03	0.00	-0.20	0.03	0.00	-20.49	3.94	0.00	-10.84	4.92	0.03	-11.57	5.55	0.04
Tertiary** (reference=Primary/secondary)	-0.18	0.04	0.00	-0.18	0.04	0.00	4.41	5.34	0.41	-0.20	0.04	0.00	-8.51	7.15	0.23	-0.20	0.04	0.00	8.45	9.24	0.36
Current smoker***	0.92	0.03	0.00	0.91	0.03	0.00	0.92	0.03	0.00	43.01	3.80	0.00	0.91	0.03	0.00	42.85	5.32	0.00	47.32	5.88	0.00
Eastern Norway****	-0.12	0.04	0.00	-0.12	0.04	0.00	-0.12	0.04	0.00	-0.11	0.04	0.01	-0.12	0.04	0.00	-0.11	0.04	0.01	-0.11	0.04	0.01
Southern/Western Norway and Trøndelag****	-0.09	0.04	0.01	-0.09	0.04	0.01	-0.09	0.04	0.01	-0.08	0.04	0.03	-0.09	0.04	0.01	-0.08	0.04	0.04	-0.08	0.04	0.04
Northern Norway****	-0.25	0.05	0.00	-0.25	0.05	0.00	-0.25	0.05	0.00	-0.24	0.05	0.00	-0.25	0.05	0.00	-0.24	0.05	0.00	-0.24	0.05	0.00
Women X Cohort				0.01	0.00	0.00							0.01	0.00	0.00	0.01	0.00	0.03	0.01	0.00	0.04
Tertiary X Cohort							0.00	0.00	0.39				0.00	0.00	0.24				0.00	0.01	0.35
Current smoker X Cohort										-0.02	0.00	0.00				-0.02	0.00	0.00	-0.02	0.00	0.00
Tertiary X Women													25.84	10.83	0.02				7.10	13.88	0.61
Tertiary X Women X Cohort													-0.01	0.01	0.02				0.00	0.01	0.61
Current smoker X Women																0.35	7.62	0.96	-4.33	8.34	0.60
Current smoker X Women X Cohort																0.00	0.00	1.00	0.00	0.00	0.58
Tertiary X Current smoker																			-30.81	14.86	0.04
Tertiary X Current smoker X Cohort																			0.02	0.01	0.04
Tertiary X Current smoker X Women																			34.91	23.27	0.13
Tertiary X Current smoker X Women X Cohort																			-0.02	0.01	0.13
Constant	64.76	6.05	0.00	72.57	6.32	0.00	63.96	6.12	0.00	46.23	6.29	0.00	73.69	6.44	0.00	52.11	6.80	0.00	50.12	7.07	0.00

* Reference = Men
** Reference = Primary/secondary
*** Reference = Non-smoker
**** Reference = Oslo and surrounding areas

**Model fit, hurdle 1**	**Number of observations**	**Log likelihood (null)**	**Log likelihood (model)**	**Degrees of freedom**	**AIC**	**BIC**
Model H1_1	31 357	-36187.2	-34090.3	30	68240.6	68491.2
Model H1_2	31 357	-36187.2	-34068.4	33	68202.8	68478.5
Model H1_3	31 357	-36187.2	-34088.6	33	68243.2	68518.8
Model H1_4	31 357	-36187.2	-34003.4	33	68072.9	68348.5
Model H1_5	31 357	-36187.2	-34057.7	42	68199.4	68550.2
Model H1_6	31 357	-36187.2	-33927.6	42	67939.2	68290.0
Model H1_7	31 357	-36187.2	-33907.7	63	67941.4	68467.7

Likelihood-ratio test for nested models

	Model H1_1	Model H1_2	Model H1_3	Model H1_4	Model H1_5	Model H1_6	Model H1_7
Model H1_1	-	0.0000	0.3337	0.0000	-	-	-
Model H1_2	-	-	-	-	0.0108	0.0000	0.0000
Model H1_3	-	-	-	-	0.0000	-	0.0000
Model H1_4	-	-	-	-	-	0.0000	0.0000
Model H1_5	-	-	-	-	-	-	0.0000
Model H1_6	-	-	-	-	-	-	0.0079
Model H1_7	-	-	-	-	-	-	-
