# Supplementary material for: Beliefs about harms of cigarette smoking among Norwegian adults born from 1899 to 1969. Do variations across education, smoking status and sex mirror the decline in smoking?
Source: PLoS One. 2022 Aug 3;17(8):e0271647. doi: 10.1371/journal.pone.0271647 (PMC9348701; doi:10.1371/journal.pone.0271647)
Supplement: S2 Table — (PDF) [file pone.0271647.s005.pdf]

S2 Table. Regression coefficients (b), standard errors (se) and p-values (p) for models included in the second hurdle (H2)

Number of CPD that can be smoked without causing harm	Model H2_1			Model H2_2			Model H2_3			Model H2_4			Model H2_5			Model H2_6			Model H2_7		
	b	se	p	b	se	p	b	se	p	b	se	p	b	se	p	b	se	p	b	se	p
Birth cohort	-0.01	0.00	0.00	-0.01	0.00	0.00	-0.01	0.00	0.00	-0.01	0.00	0.00	-0.01	0.00	0.00	-0.01	0.00	0.00	-0.01	0.00	0.00
Age	0.00	0.00	0.39	0.00	0.00	0.39	0.00	0.00	0.44	0.00	0.00	0.36	0.00	0.00	0.45	0.00	0.00	0.40	0.00	0.00	0.46
Period (Real price)	0.02	0.01	0.00	0.02	0.01	0.00	0.02	0.01	0.00	0.02	0.01	0.00	0.02	0.01	0.00	0.02	0.01	0.00	0.02	0.01	0.00
Women*	-0.24	0.02	0.00	-9.57	2.47	0.00	-0.24	0.02	0.00	-0.23	0.02	0.00	-8.04	2.66	0.00	-22.03	3.97	0.00	-20.22	4.43	0.00
Tertiary education**	-0.24	0.03	0.00	-0.24	0.03	0.00	10.79	3.78	0.00	-0.24	0.03	0.00	13.88	4.88	0.00	-0.25	0.03	0.00	14.81	6.93	0.03
Current smoker***	0.46	0.02	0.00	0.45	0.02	0.00	0.46	0.02	0.00	8.32	2.54	0.00	0.45	0.02	0.00	-1.18	3.42	0.73	0.27	3.77	0.94
Eastern Norway****	-0.09	0.03	0.00	-0.09	0.03	0.00	-0.09	0.03	0.00	-0.08	0.03	0.00	-0.09	0.03	0.00	-0.09	0.03	0.00	-0.09	0.03	0.00
Southern/Western Norway and Trøndelag****	-0.07	0.03	0.01	-0.07	0.03	0.01	-0.07	0.03	0.01	-0.07	0.03	0.01	-0.07	0.03	0.01	-0.07	0.03	0.01	-0.07	0.03	0.01
Northern Norway****	-0.13	0.04	0.00	-0.13	0.04	0.00	-0.13	0.04	0.00	-0.13	0.04	0.00	-0.13	0.04	0.00	-0.13	0.04	0.00	-0.13	0.04	0.00
Women X Cohort				0.01	0.00	0.00							0.00	0.00	0.00	0.01	0.00	0.00	0.01	0.00	0.00
Tertiary X Cohort							-0.01	0.00	0.00				-0.01	0.00	0.00				-0.01	0.00	0.03
Current smoker X Cohort										0.00	0.00	0.00				0.00	0.00	0.63	0.00	0.00	0.96
Tertiary X Women													-7.86	7.81	0.31				-4.25	11.38	0.71
Tertiary X Women X Cohort													0.00	0.00	0.31				0.00	0.01	0.71
Current smoker X Women																20.88	5.10	0.00	20.06	5.58	0.00
Current smoker X Women X Cohort																-0.01	0.00	0.00	-0.01	0.00	0.00
Tertiary X Current smoker																			-2.01	9.88	0.84
Tertiary X Current smoker X Cohort																			0.00	0.01	0.84
Tertiary X Current smoker X Women																			-4.13	15.74	0.79
Tertiary X Current smoker X Women X Cohort																			0.00	0.01	0.80
Constant	21.73	4.15	0.00	25.81	4.28	0.00	19.94	4.19	0.00	17.12	4.40	0.00	23.38	4.35	0.00	26.79	4.74	0.00	23.52	4.96	0.00
lnalpha	-0.34	0.03		-0.34	0.03		-0.34	0.03		-0.34	0.03		-0.34	0.03		-0.35	0.03		-0.35	0.03	
alpha	0.71	0.02		0.71	0.02		0.71	0.02		0.71	0.02		0.71	0.02		.71	0.02		0.71	0.02	
LR test of alpha=0: chibar2(01)		1.8e+04			1.8e+04			1.8e+04			1.8e+04			1.8e+04			1.7e+04			1.7e+04	
Prob >= chibar2		0.000			0.000			0.000			0.000			0.000			0.000			0.000	

* Reference = Men
** Reference = Primary/secondary
*** Reference = Non-smoker
**** Reference = Oslo and surrounding areas

**Model fit, hurdle 2**	**Number of observations**	**Log likelihood (null)**	**Log likelihood (model)**	**Degrees of freedom**	**AIC**	**BIC**
Model H2_1	10 614	-28626.7	-28101.6	11	56225.1	56305.1
Model H2_2	10 614	-28626.7	-28094.4	12	56212.9	56300.1
Model H2_3	10 614	-28626.7	-28097.3	12	56218.5	56305.8
Model H2_4	10 614	-28626.7	-28096.8	12	56217.5	56304.7
Model H2_5	10 614	-28626.7	-28089.5	15	56209.0	56318.0
Model H2_6	10 614	-28626.7	-28081.4	15	56192.8	56301.9
Model H2_7	10 614	-28626.7	-28076.3	22	56196.5	56356.5

Likelihood-ratio test for nested models

	Model H2_1	Model H2_2	Model H2_3	Model H2_4	Model H2_5	Model H2_6	Model H2_7
Model H2_1	-	0.0002	0.0034	0.0019	-	-	-
Model H2_2	-	-	-	-	0.0197	0.0000	0.0001
Model H2_3	-	-	-	-	0.0014	-	0.0000
Model H2_4	-	-	-	-	-	0.0000	0.0000
Model H2_5	-	-	-	-	-	-	0.0004
Model H2_6	-	-	-	-	-	-	0.1732
Model H2_7	-	-	-	-	-	-	-
