# Supplementary material for: Beliefs about harms of cigarette smoking among Norwegian adults born from 1899 to 1969. Do variations across education, smoking status and sex mirror the decline in smoking?
Source: PLoS One. 2022 Aug 3;17(8):e0271647. doi: 10.1371/journal.pone.0271647 (PMC9348701; doi:10.1371/journal.pone.0271647)
Supplement: S3 Table — (PDF) [file pone.0271647.s006.pdf]

**S3 Table. Number of respondents in each birth cohort (down) by survey year (across)**

| | 1973 | 1976 | 1977 | 1978 | 1979 | 1980 | 1981 | 1982 | 1983 | 1984 | 1985 | 1986 | 1987 | 1988 | 1989 | 1990 | 1992 | 1994 | Total |
|---|---|---|---|---|---|---|---|---|---|---|---|---|---|---|---|---|---|---|---|
| **1899** | 17 | | | | | | | | | | | | | | | | | | **17** |
| **1900** | 26 | | | | | | | | | | | | | | | | | | **26** |
| **1901** | 33 | | | | | | | | | | | | | | | | | | **33** |
| **1902** | 36 | 25 | | | | | | | | | | | | | | | | | **61** |
| **1903** | 21 | 18 | 20 | | | | | | | | | | | | | | | | **59** |
| **1904** | 29 | 24 | 17 | 14 | | | | | | | | | | | | | | | **84** |
| **1905** | 37 | 33 | 25 | 19 | 15 | | | | | | | | | | | | | | **129** |
| **1906** | 21 | 24 | 35 | 18 | 22 | 20 | | | | | | | | | | | | | **140** |
| **1907** | 41 | 27 | 32 | 29 | 30 | 28 | 17 | | | | | | | | | | | | **204** |
| **1908** | 27 | 24 | 28 | 22 | 31 | 14 | 27 | 21 | | | | | | | | | | | **194** |
| **1909** | 30 | 35 | 30 | 36 | 24 | 30 | 25 | 18 | 26 | | | | | | | | | | **254** |
| **1910** | 40 | 35 | 33 | 36 | 22 | 20 | 25 | 30 | 25 | 20 | | | | | | | | | **286** |
| **1911** | 32 | 24 | 39 | 33 | 37 | 28 | 36 | 22 | 21 | 13 | 17 | | | | | | | | **302** |
| **1912** | 43 | 38 | 33 | 31 | 32 | 37 | 32 | 23 | 25 | 23 | 21 | 22 | | | | | | | **360** |
| **1913** | 40 | 38 | 32 | 27 | 38 | 34 | 24 | 19 | 33 | 18 | 23 | 24 | 33 | | | | | | **383** |
| **1914** | 40 | 31 | 33 | 31 | 29 | 24 | 29 | 27 | 27 | 22 | 28 | 17 | 27 | 20 | | | | | **385** |
| **1915** | 43 | 30 | 30 | 39 | 38 | 26 | 30 | 31 | 22 | 33 | 41 | 29 | 29 | 19 | 16 | | | | **456** |
| **1916** | 51 | 42 | 37 | 34 | 31 | 39 | 34 | 31 | 41 | 36 | 25 | 39 | 28 | 22 | 27 | 25 | | | **542** |
| **1917** | 45 | 32 | 39 | 33 | 34 | 31 | 32 | 39 | 25 | 36 | 37 | 34 | 30 | 26 | 25 | 31 | | | **529** |
| **1918** | 42 | 31 | 34 | 41 | 45 | 33 | 37 | 29 | 24 | 36 | 27 | 41 | 30 | 42 | 26 | 28 | 21 | | **567** |
| **1919** | 42 | 40 | 34 | 42 | 39 | 39 | 32 | 36 | 43 | 28 | 37 | 22 | 24 | 25 | 31 | 25 | 15 | | **554** |
| **1920** | 42 | 46 | 42 | 62 | 41 | 35 | 28 | 44 | 50 | 27 | 33 | 37 | 49 | 36 | 33 | 39 | 32 | 19 | **695** |
| **1921** | 48 | 52 | 42 | 45 | 33 | 40 | 36 | 39 | 23 | 40 | 33 | 44 | 47 | 33 | 27 | 36 | 23 | 13 | **654** |
| **1922** | 45 | 39 | 43 | 35 | 34 | 33 | 37 | 47 | 34 | 39 | 32 | 36 | 44 | 39 | 42 | 43 | 29 | 13 | **664** |
| **1923** | 47 | 39 | 40 | 45 | 46 | 44 | 38 | 43 | 32 | 34 | 30 | 42 | 31 | 36 | 38 | 25 | 25 | 16 | **651** |
| **1924** | 37 | 49 | 32 | 36 | 41 | 33 | 41 | 30 | 28 | 26 | 38 | 31 | 32 | 31 | 30 | 39 | 19 | 22 | **595** |
| **1925** | 37 | 28 | 34 | 34 | 37 | 32 | 38 | 37 | 33 | 46 | 33 | 37 | 38 | 29 | 26 | 32 | 34 | 26 | **611** |
| **1926** | 32 | 23 | 36 | 39 | 32 | 37 | 36 | 42 | 31 | 32 | 28 | 40 | 35 | 25 | 38 | 38 | 24 | 16 | **584** |
| **1927** | 37 | 35 | 24 | 32 | 37 | 26 | 36 | 32 | 34 | 26 | 33 | 17 | 31 | 25 | 29 | 36 | 24 | 18 | **532** |
| **1928** | 29 | 34 | 31 | 26 | 40 | 32 | 32 | 26 | 26 | 38 | 34 | 35 | 27 | 35 | 31 | 22 | 18 | 26 | **542** |
| **1929** | 42 | 29 | 29 | 35 | 31 | 36 | 38 | 22 | 26 | 32 | 31 | 17 | 39 | 33 | 24 | 32 | 20 | 24 | **540** |
| **1930** | 24 | 36 | 31 | 39 | 43 | 28 | 29 | 29 | 31 | 39 | 38 | 46 | 33 | 36 | 24 | 23 | 20 | 28 | **577** |
| **1931** | 39 | 37 | 30 | 25 | 27 | 30 | 28 | 46 | 31 | 33 | 18 | 26 | 24 | 31 | 31 | 26 | 30 | 22 | **534** |
| **1932** | 37 | 39 | 28 | 29 | 24 | 27 | 31 | 19 | 32 | 28 | 23 | 23 | 29 | 23 | 29 | 35 | 19 | 18 | **493** |
| **1933** | 40 | 29 | 33 | 28 | 31 | 36 | 25 | 27 | 27 | 33 | 29 | 33 | 31 | 30 | 23 | 24 | 22 | 21 | **522** |
| **1934** | 26 | 35 | 26 | 22 | 19 | 33 | 22 | 21 | 19 | 26 | 33 | 33 | 29 | 27 | 28 | 28 | 22 | 20 | **469** |

| | | | | | | | | | | | | | | | | | | | |
|---|---|---|---|---|---|---|---|---|---|---|---|---|---|---|---|---|---|---|---|
| **1935** | 28 | 30 | 28 | 28 | 25 | 27 | 34 | 34 | 21 | 32 | 26 | 25 | 19 | 21 | 19 | 32 | 20 | 26 | **475** |
| **1936** | 41 | 30 | 16 | 31 | 35 | 37 | 34 | 36 | 28 | 27 | 39 | 27 | 28 | 34 | 32 | 24 | 22 | 24 | **545** |
| **1937** | 22 | 32 | 34 | 35 | 31 | 31 | 22 | 24 | 28 | 13 | 20 | 26 | 34 | 25 | 31 | 20 | 20 | 15 | **463** |
| **1938** | 29 | 26 | 25 | 37 | 44 | 27 | 29 | 44 | 31 | 29 | 31 | 29 | 48 | 25 | 30 | 29 | 16 | 20 | **549** |
| **1939** | 27 | 38 | 30 | 34 | 47 | 30 | 17 | 31 | 23 | 34 | 31 | 42 | 47 | 25 | 36 | 32 | 24 | 23 | **571** |
| **1940** | 24 | 28 | 39 | 34 | 37 | 29 | 29 | 28 | 27 | 28 | 31 | 23 | 38 | 32 | 28 | 42 | 19 | 24 | **540** |
| **1941** | 29 | 40 | 34 | 25 | 31 | 31 | 31 | 36 | 38 | 34 | 29 | 43 | 37 | 34 | 39 | 39 | 18 | 20 | **588** |
| **1942** | 40 | 37 | 30 | 30 | 44 | 27 | 35 | 29 | 42 | 39 | 42 | 43 | 44 | 24 | 38 | 29 | 26 | 31 | **630** |
| **1943** | 31 | 41 | 31 | 38 | 34 | 31 | 38 | 40 | 41 | 41 | 38 | 33 | 46 | 28 | 49 | 49 | 32 | 26 | **667** |
| **1944** | 29 | 38 | 43 | 43 | 50 | 38 | 42 | 46 | 32 | 40 | 39 | 33 | 44 | 60 | 37 | 37 | 27 | 39 | **717** |
| **1945** | 35 | 41 | 46 | 46 | 42 | 53 | 41 | 53 | 43 | 38 | 42 | 41 | 57 | 48 | 49 | 44 | 39 | 41 | **799** |
| **1946** | 36 | 44 | 46 | 42 | 58 | 46 | 51 | 48 | 54 | 47 | 40 | 63 | 49 | 55 | 47 | 37 | 42 | 35 | **840** |
| **1947** | 41 | 43 | 39 | 39 | 45 | 47 | 34 | 53 | 40 | 38 | 41 | 50 | 43 | 72 | 48 | 49 | 42 | 26 | **790** |
| **1948** | 31 | 46 | 35 | 40 | 51 | 38 | 46 | 46 | 45 | 54 | 43 | 53 | 58 | 45 | 47 | 57 | 48 | 37 | **820** |
| **1949** | | 28 | 38 | 35 | 47 | 43 | 52 | 52 | 30 | 46 | 40 | 43 | 54 | 54 | 46 | 46 | 39 | 34 | **727** |
| **1950** | | 26 | 41 | 51 | 33 | 48 | 37 | 41 | 46 | 41 | 49 | 54 | 57 | 44 | 51 | 49 | 46 | 35 | **749** |
| **1951** | | 41 | 29 | 32 | 48 | 44 | 39 | 26 | 39 | 49 | 57 | 42 | 51 | 57 | 45 | 56 | 31 | 41 | **727** |
| **1952** | | | 34 | 36 | 37 | 34 | 51 | 38 | 31 | 45 | 41 | 51 | 59 | 31 | 53 | 44 | 39 | 45 | **669** |
| **1953** | | | | 32 | 34 | 36 | 42 | 49 | 49 | 45 | 30 | 49 | 45 | 40 | 44 | 49 | 29 | 43 | **616** |
| **1954** | | | | | 33 | 38 | 48 | 31 | 43 | 52 | 49 | 51 | 43 | 68 | 45 | 50 | 36 | 39 | **626** |
| **1955** | | | | | | 44 | 36 | 41 | 54 | 42 | 51 | 47 | 48 | 48 | 49 | 30 | 48 | 41 | **579** |
| **1956** | | | | | | | 34 | 40 | 29 | 39 | 39 | 48 | 42 | 46 | 54 | 57 | 38 | 39 | **505** |
| **1957** | | | | | | | | 43 | 36 | 43 | 56 | 60 | 55 | 47 | 60 | 39 | 45 | 35 | **519** |
| **1958** | | | | | | | | | 30 | 52 | 45 | 41 | 37 | 50 | 46 | 45 | 45 | 48 | **439** |
| **1959** | | | | | | | | | | 36 | 43 | 43 | 53 | 48 | 39 | 39 | 38 | 38 | **377** |
| **1960** | | | | | | | | | | | 32 | 52 | 60 | 37 | 33 | 45 | 43 | 34 | **336** |
| **1961** | | | | | | | | | | | | 49 | 54 | 34 | 41 | 51 | 30 | 40 | **299** |
| **1962** | | | | | | | | | | | | | 54 | 28 | 48 | 45 | 37 | 44 | **256** |
| **1963** | | | | | | | | | | | | | | 36 | 48 | 44 | 40 | 45 | **213** |
| **1964** | | | | | | | | | | | | | | | 44 | 41 | 33 | 41 | **159** |
| **1965** | | | | | | | | | | | | | | | | 42 | 34 | 35 | **111** |
| **1966** | | | | | | | | | | | | | | | | | 33 | 34 | **67** |
| **1967** | | | | | | | | | | | | | | | | | 32 | 43 | **75** |
| **1968** | | | | | | | | | | | | | | | | | | 47 | **47** |
| **1969** | | | | | | | | | | | | | | | | | | 39 | **39** |
| **Total** | **1,741** | **1,710** | **1,650** | **1,705** | **1,789** | **1,684** | **1,697** | **1,739** | **1,649** | **1,748** | **1,746** | **1,886** | **2,024** | **1,819** | **1,854** | **1,879** | **1,508** | **1,529** | **31,357** |
